# Investigating Individual- and Area-Level Socioeconomic Gradients of Pulse Pressure among Normotensive and Hypertensive Participants

**DOI:** 10.3390/ijerph10020571

**Published:** 2013-02-04

**Authors:** Lisa A. Matricciani, Catherine Paquet, Natasha J. Howard, Robert Adams, Neil T. Coffee, Anne W. Taylor, Mark Daniel

**Affiliations:** 1 Social Epidemiology and Evaluation Research Group, Sansom Institute for Health Research, School of Population Health, Division of Health Sciences, University of South Australia, Adelaide 5001, Australia; E-Mails: matla005@mymail.unisa.edu.au (L.A.M.); catherine.paquet@unisa.edu.au (C.P.); natasha.howard@unisa.edu.au (N.J.H.); neil.coffee@unisa.edu.au (N.T.C.);; 2 Research Centre of the Douglas Mental Health University Institute, Verdun, Quebec H4H 1R2, Canada; 3 The Health Observatory, Discipline of Medicine, The University of Adelaide, Adelaide, South Australia 5005, Australia; E-Mail: robert.adams@adelaide.edu.au; 4 Population Research and Outcome Studies, Discipline of Medicine, The University of Adelaide, Adelaide, South Australia 5005, Australia; E-Mail: anne.taylor@adelaide.edu.au; 5 Department of Medicine, The University of Melbourne, St Vincent’s Hospital, Melbourne, Victoria 3065, Australia

**Keywords:** pulse pressure, socioeconomic status, residence characteristics, geographic information system, education, income, employment

## Abstract

Socioeconomic status is a strong predictor of cardiovascular disease. Pulse pressure, the difference between systolic and diastolic blood pressure, has been identified as an important predictor of cardiovascular risk even after accounting for absolute measures of blood pressure. However, little is known about the social determinants of pulse pressure. The aim of this study was to examine individual- and area-level socioeconomic gradients of pulse pressure in a sample of 2,789 Australian adults. Using data from the North West Adelaide Health Study we estimated the association between pulse pressure and three indices of socioeconomic status (education, income and employment status) at the area and individual level for hypertensive and normotensive participants, using Generalized Estimating Equations. In normotensive individuals, area-level education (estimate: −0.106; 95% CI: −0.172, −0.041) and individual-level income (estimate: −1.204; 95% CI: −2.357, −0.050) and employment status (estimate: −1.971; 95% CI: −2.894, −1.048) were significant predictors of pulse pressure, even after accounting for the use of medication and lifestyle behaviors. In hypertensive individuals, only individual-level measures of socioeconomic status were significant predictors of pulse pressure (education estimate: −2.618; 95% CI: −4.878, −0.357; income estimate: −1.683, 95% CI: −3.743, 0.377; employment estimate: −2.023; 95% CI: −3.721, −0.326). Further research is needed to better understand *how* individual- and area-level socioeconomic status influences pulse pressure in normotensive and hypertensive individuals.

## 1. Introduction

There is substantial evidence that cardiovascular disease (CVD) is strongly associated with socioeconomic status, expressed in terms of income, education and/or occupational status [1,2]. Both prospective [3,4] and cross-sectional [5,6,7,8] studies have shown that low socioeconomic status is associated with elevated cardiovascular risk in men and women, across different countries. Although studies have traditionally examined socioeconomic status at the individual level, socioeconomic status at the area level is increasingly being recognized as a strong predictor of CVD, independent of individual-level socioeconomic status [9,10,11]. These findings reflect the way in which area-level socioeconomic status, unlike individual-level socioeconomic status, accounts for the geographic dispersion of individual and contextual risk factors. Consideration of social circumstance at both the individual and area level has recently been recommended [12] when assessing socioeconomic status variations in cardiovascular risk factors, such as blood pressure, one of the strongest known cardiovascular risk factors [13,14,15,16,17].

Evaluating socioeconomic status differences in blood pressure could help explain the social gradient that exists for CVD. Kaplan and Keil [2] in a narrative literature review identified a consistent inverse association between individual-level socioeconomic status and hypertension across identified studies while Nogueira and colleagues [18], in a multicenter collaborative study involving seven countries, demonstrated that low individual-level socioeconomic status was associated with elevated blood pressure. More recently, Avendano and colleagues [19] in a longitudinal study of 10 European countries identified low individual-level socioeconomic status was associated with an increased risk of cardiovascular-related mortality. Similar findings have also been demonstrated for area-level socioeconomic status [20]. Chaix and colleagues [21] reporting on the PRIME Study, documented a strong monotonic increase in blood pressure as associated with decreasing area-level education. Cozier and colleagues [22], in a study of 36,099 women, reported a significant inverse association between hypertension and area-level income in analyses accounting for individual-level socioeconomic status. 

Although hypertension and systolic blood pressure are widely recognized as important predictors of cardiovascular risk [23,24], an emerging evidence base suggests that pulse pressure, the difference between systolic and diastolic blood pressure, may be an important independent predictor of CVD, even after accounting for absolute measures of blood pressure [25,26,27]. Panagiotakos and colleagues [28], in a seven-country prospective study of 12,763 people living in the United States, Japan, Italy, Greece, Finland, former Yugoslavia and The Netherlands, identified pulse pressure, followed by diastolic and systolic blood pressure, as the best predictor for CVD mortality over the twenty-five-year follow up period.

Given the well documented inverse association between socioeconomic status and hypertension, it might be assumed that pulse pressure would also vary according to socioeconomic status. However, such an association has not been established. Regidor and colleagues [29] in a representative study of 4,009 Spaniards, found no association between childhood socioeconomic status and pulse pressure in adulthood, while Banegas and colleagues [30], examining the same population, observed that socioeconomic status, expressed as educational attainment, was significantly inversely associated with pulse pressure in hypertensive individuals. Rogers and Saint Onge [31] in a study of 16,532 adults identified measures of socioeconomic status explained pulse pressure differences between males and females and by racial/ethnic groups. While few studies have examined the association between pulse pressure and socioeconomic status at the individual level, it appears that no study has yet reported an examination of associations between pulse pressure and different measures of area-level socioeconomic status. Such information is needed to determine whether pulse pressure is affected similarly or differently from blood pressure in relation to area-level socioeconomic status.

On the basis that pulse pressure is strongly correlated with systolic blood pressure [28,32,33], it is possible that pulse pressure, like systolic blood pressure, is strongly inversely associated with socioeconomic status. On the other hand, this may not be the case. For example, an individual might have an elevated systolic blood pressure, but may or may not have a widened pulse pressure (e.g., blood pressure = 150/90, pulse pressure = 60 mmHg, *vs.* blood pressure = 150/110, pulse pressure = 40 mmHg). Similarly, an individual might have a widened pulse pressure, but may or may not be hypertensive (e.g., blood pressure = 190/130, pulse pressure = 60 mmHg, *vs.* blood pressure = 125/65, pulse pressure = 60 mmHg). It is unclear whether an association between socioeconomic status and pulse pressure is similar to or different from documented associations between socioeconomic status and blood pressure status.

Several studies have examined the importance of pulse pressure in relation to hypertensive state. Benetos and colleagues [34], in a prospective study of 19,083 men, found that a widened pulse pressure was a strong predictor of myocardial infarction in both normotensive and hypertensive individuals. Other studies [35,36] have suggested that pulse pressure may be a more important predictor of cardiovascular risk in individuals with normal systolic blood pressure (<140) and diastolic blood pressure (<90) than in hypertensive individuals. These studies are interesting, given that death from CVD in treated hypertensive subjects (*i.e.*, normotensive) accounts for approximately 68% of all deaths [36].

To date, no published study, to our knowledge, has reported on the associations between socioeconomic status and pulse pressure at both the individual and area level. Nor has any published study reported on the association between socioeconomic status and pulse pressure as this may vary according to hypertensive state (*i.e.*, normotensive *vs.* hypertensive). Understanding how socioeconomic status is associated with pulse pressure may provide important insight that will help us better understand the social gradients that exist for cardiovascular risk and in turn, help inform public policies, treatments, guidelines and interventions.

## 2. Methods

### 2.1. Study Context

This study was conducted as a part of the Place and Metabolic Syndrome (PAMS) project which links and evaluates population-based health information in relation to local community characteristics hypothesized to be associated with markers of metabolic syndrome. The PAMS project expands on the North West Adelaide Health Study (NWAHS), a longitudinal study of chronic conditions and health-related risk factors [37]. Individual-level data have been assigned a geo-reference based on residential address at the time of data collection, allowing participants to be spatially referenced using a geographic information system (GIS). Participants were recruited from the northern and western region of metropolitan Adelaide, a region that reflects 38% of the metropolitan region of Adelaide and 28% of the state’s population [38]. The socio-demographic profile of the NWAHS is comparable to the metropolitan region of Adelaide in terms of education and income, with 11.3% of participants having a bachelor’s degree and 69.4% of households reporting earnings of over 20,000 AUD each year. The PAMS project holds ethics approvals from Human Research Ethics Committees at the University of South Australia, Central Northern Adelaide Health Service, and South Australian Department of Health.

### 2.2. Sampling

Thus far, the NWAHS has collected three waves of data over a ten-year period. Wave 1 data collection was conducted between January 2000 and June 2003, Wave 2 of data collection was conducted between May 2004 and February 2006 and Wave 3 of data collection was conducted between June 2008 and June 2010 [37]. This analysis utilized only Wave 2 information as this series of data includes current prescribed hypertensive medication extracted from the Australian Pharmaceutical Benefits Scheme for all participants. 

Individuals were eligible to participate in the NWAHS study if, at the time of recruitment, they were 18 years or older and resided within a household in the north-west region of Adelaide, South Australia, with a telephone connected and a telephone number listed in the Electronic White Pages (EWP) telephone directory [39]. Households were randomly selected from the EWP. The resident having had the most recent birthday and aged 18 years or older was invited to participate. Enrolled participants who subsequently changed residential location remained eligible for follow-up data collections. Of the 8,213 eligible individuals who agreed to take part in the study, 5,850 completed an interview and 4,056 attended a clinic assessment. Of the Wave 1 participants, 3,564 completed a Wave 2 follow-up questionnaire, and 3,206 attended a follow-up clinic assessment. 

### 2.3. Data Collection

Participants who agreed to take part in the study were required to sit a computer-assisted telephone interview, complete a self-administered questionnaire and attend a clinical assessment where blood pressure measurements were collected. Hypertension was defined according to blood pressure limits set by the International Diabetes Federation (IDF) definition, corresponding to systolic blood pressure greater than or equal to 130 mmHg or diastolic blood pressure greater than or equal to 85 mmHg [40]. Information on current medications prescribed for each participant was obtained by linking Australian Pharmaceutical Benefits Scheme data to the participant’s Medicare number.

### 2.4. Measures

#### 2.4.1. Dependent Variables

The dependent variable was a continuous pulse pressure score. Pulse pressure was calculated as the difference between the average of two systolic and diastolic blood pressure measures, which were assessed by a trained clinic research assistant according to the Standard Operating Procedures for the Australian National Blood Pressure Study [41]. Readings were taken using a manual sphygmomanometer after participants were seated for a minimum of three minutes of quiet rest. Blood pressure cuff size was selected based on arm circumference. Systolic blood pressure was recorded at the first appearance (Phase I) of Korotkoff sounds, and diastolic blood pressure was recorded at the disappearance (Phase V) of Korotkoff sounds. Pulse pressures >10 mmHg were included for analysis.

#### 2.4.2. Independent Variables

Independent variables were individual- and area-level measures of socioeconomic status pertaining to education, income and employment status.

##### Area-Level Measures of Socioeconomic Status

All area-level measures were obtained from the Australian Bureau of Statistics (ABS) 2006 Population and Housing Census, defined at the State Suburb level. State Suburbs are Census Geographic Units formed by aggregating Census Collection Districts to approximate the locality (suburb) boundaries within urban areas [42]. The selection of the State Suburb ensured a sufficient number of participants per spatial unit as well as larger between-unit variability. *Education* was expressed as the proportion of residents within the State Suburb with a bachelor’s degree or higher. *Income* was defined as the median household income within the defined spatial unit and *employment* status was recorded as the proportion of residents over the age of 15 years, within the State Suburb, in the workforce.

##### Individual-Level Measures of Socioeconomic Status

All individual-level measures of socioeconomic status were collected via self-administered questionnaire. *Educational* attainment was recorded as whether the participant had a bachelor’s degree, or not. *Income* was recorded as annual gross household income, categorized as: <20,000 AUD; 20,000–60,000 AUD or >60,000 AUD. *Employment* status was defined according to whether the participant was “currently in the workforce” (*i.e.*, either full time, or part time/casual employment) or ‘not currently in the workforce’ (*i.e.*, either unemployed, student, homemaker, retired, or other).

###### 2.4.3. Covariates

Covariates included participant’s age, sex, height, use of prescribed medications, and lifestyle behaviors including physical activity, alcohol consumption and smoking status. These variables were used on the basis of previous research [43,44,45,46,47,48] that identified associations between these variables and both pulse pressure and socioeconomic status. Weight was not included as a covariate as the association between pulse pressure and weight status remains unclear [29,45]. Participant’s *age* was entered as a continuous variable and *sex* was entered as a categorical variable. *Height* was assessed without shoes using a wall-mounted stadiometer and was recorded as a continuous variable to the nearest 0.5 centimeters. Participants were categorized as using *prescribed medication* if they had a prescription filled within the last six months for any hypertensive medication included on the Australian Pharmaceutical Benefits Scheme. This includes medications classified as diuretics, beta-blockers, anti-hypertensive agents, calcium-channel blockers and renin-angiotensin agents. Level of *physical activity* was determined from questions previously employed within the ABS, National Health Survey [49] and expressed as Metabolic Equivalence Tasks (METs), in hours per week, derived from the total amount and intensity of physical activity (walking, moderate and vigorous physical activity) undertaken for sport, recreation or fitness within the last two weeks [50]. Participants were assigned to either the “low-sedentary” category if they achieved less than 1,600 METs, or to the “moderate-high” physical activity group if they achieved 1,600 METs or more, or engaged in more than two hours of vigorous exercise in the two weeks preceding survey completion. *Smoking status* questionnaire data was coded as either a “smoker” (*i.e.*, current smoker) or a “non-smoker” (*i.e.*, either never smoked, or ex-smoker). *Alcohol consumption* was coded using national guidelines [51] according to “at risk” or “not at risk” in terms of excessive alcohol consumption. Male participants were coded “at risk” if they consumed more than six standard drinks on any one day, an average of more than four drinks per day, or more than 28 standard drinks over a week. Female participants were coded “at risk” if they consumed more than four standard drinks on any one day, an average of more than two drinks per day, or more than 14 standard drinks over a week.

### 2.5. Statistical Analysis

Generalized Estimating Equations (GEE) linear models were used to estimate the associations between pulse pressure and three separate measures of socioeconomic status (education, income and employment status) at the individual and area level. All analyses were estimated using GEE as it provides a method of estimating population-averaged effects that accounts for spatial clustering. This method has been recommended over random effects model for studies on neighbourhood influences on health [52]. Analyses were conducted according to hypertensive state (*i.e.*, normotensive and hypertensive). For each of the three socioeconomic measures, three separate regression models were fitted to assess the association between pulse pressure and the given socioeconomic status measure. Model 1 included the individual-level socioeconomic status measure and its corresponding area-level analogue as independent variables and covariates including age, sex and height. Model 2 included use of prescribed medication as an additional covariate. Model 3 included use of prescribed medication as well as lifestyle behaviors (physical activity, smoking status, alcohol consumption) as additional covariates. Statistical analyses were performed with SAS 9.2 System for Windows (SAS Institute, Cary, NC, USA).

## 3. Results

### 3.1. Sample Characteristics

Of the 3,206 participants who attended a follow-up clinic assessment and completed the Wave 2 questionnaire, 3,155 participants had a recorded pulse pressure and geo-reference. Of these, 2,977 participants had complete data on all independent variables examined, of whom, one participant had missing data on height and 187 participants had missing data on lifestyle behaviors (physical activity, alcohol consumption, smoking status). Thus, a total of 2,789 participants had complete data on all required variables and were included for analysis. The number of people living within the average spatial unit was 4,804 (range: 120–23,493).

The mean age of participants was 55 years (age range: 22–94 years) with mean pulse pressure 47 mmHg (pulse pressure range 13 mmHg–133 mmHg). Overall, there were approximately equal proportion of males and females, with a greater number of people being classified as low or medium income earners and not holding a bachelor’s degree. There were an approximately equal number of people in the workforce as there were not in the workforce. Characteristics of the sample analysed are shown in Table 1. In general, participants with hypertension were older, male and had a higher pulse pressure. Participants who were hypertensive were also more likely to be living in a low income household, not hold a bachelor’s degree, not be in the workforce and were less likely to smoke.

**Table 1:** Sample characteristics (n = 2,789).

Characteristics	Normotensive	Hypertensive	*p*-value ^ǂ^
	(n = 1,352)	(n = 1,437)	
Age (years) (mean (SD))	48.54 (14.6)	60.63 (14.0)	<0.0001
Males (% male)	539 (40%)	810 (56%)	<0.0001
Height (centimeters) (mean (SD))	168.1 (9.3)	167.9 (10.2)	0.532
Pulse pressure (mmHg) (mean (SD))	39. 3 (7.7)	54.0 (15.7)	<0.0001
Low household income (n%)	248 (18%)	478 (33%)	
Medium household income (n%)	626 (46%)	676 (47%)	<0.0001
High household income (n%)	478 (36%)	283 (20%)	
Bachelor degree (n% with a degree)	251 (19%)	119 (0.1%)	<0.0001
Employment status (n% in the workforce)	914 (68%)	631 (44%)	<0.0001
Medication (n% taking medication)	139 (10%)	573 (40%)	<0.0001
Physical activity (n% low-sedentary)	909 (67%)	1006 (70%)	0.115
Smoke status (n% smoke)	261 (19%)	164 (11%)	<0.0001
Alcohol (n% at risk)	351 (26%)	356 (25%)	0.471

**^ǂ^** Chi-squared tests were used to test for differences in categorical variables and Student’s *t*-tests were used to test for differences in continuous variables. Note: SD = standard deviation.

### 3.2. Pulse Pressure and Education

Table 2 presents the relationship between pulse pressure and education status, according to hypertensive state, for statistical Models 1 to 3. Area-level education was a strong and statistically significant predictor of pulse pressure in normotensive individuals, while individual-level education was a strong and statistically significant predictor of pulse pressure in hypertensive individuals. These associations persisted even after accounting for the use of medication and lifestyle behaviors.

### 3.3. Pulse Pressure and Income

Table 3 presents associations identified between pulse pressure and income for hypertensive and normotensive individuals. As shown, low individual income level (compared to high income level) was associated with a higher pulse pressure for statistical Models 1 and 3. Area-level income was not a significant predictor of pulse pressure in normotensive or hypertensive individuals. 

### 3.4. Pulse Pressure and Employment

As presented in Table 4, individual-level employment was a statistically significant and strong predictor of pulse pressure for normotensive as well hypertensive individuals, even after accounting for additional covariates. Area-level employment was not related to pulse pressure in normotensive or hypertensive individuals.

## 4. Discussion

Socioeconomic status is a powerful and complex determinant of health. This study examined the associations between pulse pressure and individual- and area-level measures of socioeconomic status, according to hypertensive status. In our sample of 2,789 Australian adults, area-level education and individual-level income and employment status were significant predictors of pulse pressure in normotensive participants, while only individual-level measures of socioeconomic status were significant predictors of pulse pressure in hypertensive participants.

Our findings are consistent with previous studies that examine the association between pulse pressure and individual-level education. Banegas and colleagues [30] examined the association between pulse pressure and education in hypertensive participants in a study that assessed the prevalence, awareness and control of hypertension among elderly people in Spain. In this study, an inverse association between pulse pressure and educational attainment was identified with higher levels of educational attainment being associated with lower pulse pressure. The authors of this study noted that participants with lower levels of education had higher levels of treatment and poorer control of hypertension. In our study, we similarly identified a significant association between pulse pressure and individual-level education in hypertensive individuals. Interestingly, this association weakened after accounting for the use of prescribed medication and lifestyle behaviors, possibly suggesting that education influences the use of medication, as well as lifestyle behaviors, thereby influencing pulse pressure in hypertensive individuals. Indeed, previous studies have identified associations between educational attainment, medication compliance and lifestyle behavior, whereby individuals with high levels of education are more likely to receive antihypertensive treatment and exhibit greater blood pressure control, and engage in more physical activity, and less likely to smoke cigarettes, compared to individuals with lower levels of education [53,54,55,56,57,58]. Direct comparisons of results for income and employment are not possible given the paucity of studies on this topic. Other studies, described in a review by Colhoun and colleagues [59], have, however, identified significant associations between systolic blood pressure and measures of individual-level income and employment status, whereby low-income earners and unemployed individuals are more likely to have elevated systolic blood pressure.

Similarly, while there do not appear to be any prior published studies that have examined the associations between pulse pressure and different area-level measures of socioeconomic status, previous studies [21,22,60] that have examined the association between hypertension and area-level measures of socioeconomic status have identified strong associations, even after adjusting for individual-level socioeconomic status and other potential confounders. In our study, area-level education was a statistically significant, strong predictor of pulse pressure in normotensive subjects, even after accounting for individual-level education, use of medication, and lifestyle behaviors. This finding suggests that area-level education influences pulse pressure over and above individual characteristics, depending on hypertensive status. Further efforts are needed to better understand the mechanisms underlying this association.

It is likely that associations involving socioeconomic status reflect underlying, unmeasured, risk factors or determinants of health rather than any direct influence of socioeconomic status itself. However, the mechanisms by which social status affects health are still unclear. Disparities in social conditions such as education, income and employment status are thought to reflect underlying inequalities in access to, and utilization of, social support structures and health care systems [61,62]. For instance, education has been suggested to influence health by providing knowledge and life skills that equip individuals with the ability to gain access to information and resources that promote health, while income is thought to influence health by providing means of purchasing health care and providing better nutrition, housing and schooling [63,64]. Employment status, on the other hand, is believed to influence health by reducing financial strain, poverty and psychological distress as well as increasing self-esteem and general health [64,65,66]. However, some of these associations may be related to the “healthy worker” effect, that is, employed individuals may exhibit better health than the general population since individuals who are severely ill and/or chronically disabled are generally excluded from employment [67].

Similarly, area-level socioeconomic status is thought to influence health through features of the physical or built and social environments, including the availability and accessibility of services, via multiple pathways [62,68]. For instance, area-level education is thought to be related to community engagement and social norms, which in turn, may influence lifestyle behaviors, while area-level income is thought to be related to the built environment and available services, thereby influencing health. For example, low-income areas have been associated with a higher density of fast-food restaurants [69,70,71] and fewer opportunities for physical activity (e.g., fewer parks, unsafe streets and playgroups) [72,73]. Area-level unemployment, on the other hand, is thought to contribute towards social disorganisation, leading to an increase in crime and encouraging social isolation, which in turn may influence health via stress pathways [74].

**Table 2:** Results of models testing the associations between pulse pressure and education attainment, according to hypertensive state.

	Model 1			Model 2			Model 3		
	Estimate	95% CI	*p*-value	Estimate	95% CI	*p*-value	Estimate	95% CI	*p*-value
*Normotensive participants*									
Male (*vs*. female)	0.796	−0.261; 1.852	0.140	0.821	−0.237; 1.879	0.128	0.849	−0.223; 1.920	0.121
Age	0.172	0.142; 0.202	<0.0001	0.150	0.119; 0.181	<0.0001	0.149	0.118; 0.181	<0.0001
Bachelor’s degree (*vs.* no degree)	−0.537	−1.528; 0.454	0.288	−0.531	−1.518; 0.457	0.292	−0.598	−1.603; 0.407	0.243
Area-level education	−0.110	−0.177; −0.043	0.001	−0.103	−0.170; −0.037	0.002	−0.106	−0.172; −0.041	0.002
Height	−0.045	−0.107; 0.018	0.160	−0.043	−0.106; 0.019	0.176	−0.043	−0.105; 0.019	0.173
Use of medication (*vs.* no medication)				2.613	1.093; 4.132	0.001	2.588	1.069; 4.108	0.001
High physical activity (high *vs.* low)							0.034	−0.801; 0.869	0.937
Smoker (*vs.* non-smoker)							−0.585	−1.548; 0.378	0.234
“At risk” alcohol consumption (*vs*. not at risk)							0.318	−0.490; 1.126	0.440
*Hypertensive participants*									
Male (*vs*. female)	−0.067	−2.055; 1.921	0.947	0.201	−1.832; 2.233	0.847	0.155	−1.902; 2.213	0.883
Age	0.658	0.611; 0.704	<0.0001	0.610	0.556; 0.664	<0.0001	0.619	0.565; 0.674	<0.0001
Bachelor’s degree (*vs.* no degree)	−3.038	−5.230; −0.846	0.007	−2.848	−5.066; −0.629	0.012	−2.618	−4.878; −0.357	0.023
Area-level education	−0.013	−0.133; 0.108	0.838	0.007	−0.115; 0.128	0.917	0.017	−0.106; 0.139	0.790
Height	−0.058	−0.154; 0.038	0.233	−0.065	−0.161; 0.032	0.191	−0.064	−0.160; 0.032	0.191
Use of medication (*vs.* no medication)				3.399	1.941; 4.857	<0.0001	3.432	1.985; 4.879	<0.0001
High physical activity (high *vs.* low)							0.024	−1.241; 1.289	0.970
Smoker (*vs.* non-smoker)							2.697	0.780; 4.615	0.006
“At risk” alcohol consumption (*vs*. not at risk)							−0.540	−2.262; 1.183	0.539

**Table 3:** Results of models testing the associations between pulse pressure and household income, according to hypertensive state.

	Model 1			Model 2			Model 3		
	Estimate	95% CI	*p*-value	Estimate	95% CI	*p*-value	Estimate	95% CI	*p*-value
*Normotensive participants*									
Male (*vs.* female)	0.866	−0.179; 1.911	0.104	0.889	−0.158; 1.936	0.096	0.923	−0.135; 1.981	0.087
Age	0.161	0.131; 0.191	<0.0001	0.143	0.113; 0.174	<0.0001	0.143	0.111; 0.174	<0.0001
Income (high *vs.* low )	−1.485	−2.646;−0.325	0.012	−1.168	−2.342; 0.007	0.052	−1.204	−2.357; −0.050	0.041
Income (high *vs.* middle)	−0.233	−1.092; 0.627	0.595	−0.136	−0.979; 0.708	0.752	−0.173	−1.019; 0.673	0.689
Area-level income	−0.002	−0.004; 0.000	0.085	−0.002	−0.004; 0.000	0.116	−0.002	−0.004; 0.000	0.102
Height	−0.047	−0.109; 0.015	0.140	−0.047	−0.109; 0.016	0.144	−0.046	−0.108; 0.016	0.146
Use of medication (*vs.* no medication)				2.456	0.913; 3.998	0.002	2.419	0.882; 3.957	0.002
High physical activity (high *vs.* low)							0.201	−0.642; 1.043	0.641
Smoker (*vs.* non-smoker)							−0.502	−1.434; 0.429	0.290
“At risk” alcohol consumption (*vs.* not at risk)							0.269	−0.537; 1.075	0.513
*Hypertensive participants*									
Male (*vs.* female)	−0.001	−1.997; 1.994	0.999	0.258	−1.774; 2.291	0.803	0.192	−1.872; 2.256	0.855
Age	0.633	0.582; 0.684	<0.0001	0.591	0.534; 0.649	<0.0001	0.605	0.546; 0.663	<0.0001
Income (high *vs.* low)	−2.397	−4.441;−0.354	0.022	−2.004	−4.015; 0.006	0.051	−1.683	−3.743; 0.377	0.109
Income (high *vs.* middle)	−1.009	−2.686; 0.667	0.238	−0.823	−2.491; 0.845	0.334	−0.725	−2.391; 0.941	0.394
Area-level income	−0.001	−0.005; 0.002	0.433	−0.001	−0.004; 0.002	0.543	−0.001	−0.004; 0.003	0.596
Height	−0.053	−0.148; 0.042	0.271	−0.061	−0.156; 0.035	0.214	−0.061	−0.156; 0.033	0.205
Use of medication (*vs.* no medication)				3.324	1.870; 4.777	<0.0001	3.366	1.926; 4.806	<0.0001
High physical activity (high *vs.* low)							0.008	−1.252; 1.268	0.990
Smoker (*vs.* non-smoker)							2.647	0.693; 4.601	0.008
“At risk” alcohol consumption (*vs.* not at risk)							−0.369	−2.114; 1.377	0.679

**Table 4:** Results of models testing the associations between pulse pressure and employment status, according to hypertensive state.

	Model 1			Model 2			Model 3		
	Estimate	95% CI	*p*-value	Estimate	95% CI	*p*-value	Estimate	95% CI	*p*-value
*Normotensive participants*									
Sex	0.957	−0.105; 2.018	0.077	0.963	−0.100; 2.025	0.076	1.002	−0.076; 2.080	0.069
Age	0.141	0.108; 0.174	<0.0001	0.125	0.092; 0.158	<0.0001	0.126	0.092; 0.159	<0.0001
In the workforce (*vs.* not in the workforce)	−2.193	−3.087; −1.298	<0.0001	−1.953	−2.871; −1.035	<0.0001	−1.971	−2.894; −1.048	<0.0001
Area-level employment	0.149	−0.138; 0.436	0.308	0.135	−0.163; 0.433	0.375	0.131	−0.166; 0.428	0.387
Height	−0.050	−0.112; 0.013	0.117	−0.049	−0.112; 0.014	0.129	−0.048	−0.111; 0.014	0.129
Use of medication (*vs.* no medication)				2.324	0.795; 3.854	0.003	2.301	0.775; 3.828	0.003
High physical activity (high *vs.* low)							0.227	−0.610; 1.065	0.595
Smoker (*vs.* non-smoker)							−0.380	−1.316; 0.555	0.426
“At risk” alcohol consumption (*vs.* not at risk)							0.343	−0.475; 1.161	0.411
*Hypertensive participants*									
Sex	0.307	−1.732; 2.347	0.768	0.484	−1.582; 2.549	0.646	0.411	−1.689; 2.511	0.701
Age	0.600	0.538; 0.661	<0.0001	0.571	0.506; 0.636	<0.0001	0.582	0.515; 0.649	<0.0001
In the workforce (*vs.* not in the workforce)	−2.797	−4.455; −1.140	0.001	−2.099	−3.745; −0.453	0.012	−2.023	−3.721; −0.326	0.020
Area-level employment	−0.055	−0.587; 0.478	0.841	−0.068	−0.591; 0.455	0.800	−0.100	−0.617; 0.418	0.706
Height	−0.065	−0.159; 0.029	0.177	−0.071	−0.166; 0.025	0.147	−0.069	−0.162; 0.025	0.152
Use of medication (*vs.* no medication)				3.187	1.744; 4.630	<0.0001	3.211	1.785; 4.637	<0.0001
High physical activity (high *vs.* low)							0.159	−1.125; 1.444	0.808
Smoker (*vs.* non-smoker)							2.822	0.907; 4.737	0.004
“At risk” alcohol consumption (*vs.* not at risk)							−0.411	−2.135; 1.314	0.641

In this study, individual-level education, income and employment status were each statistically significant predictors of pulse pressure in hypertensive participants, whereas area-level education and individual-level income and employment status were significant predictors of pulse pressure in normotensive participants. Area-level education may thus offer protection against cardiovascular risk in normotensive individuals, over and above individual characteristics, by protecting against elevated pulse pressure. Such findings may provide important insight in the development of primary, secondary and tertiary interventions that aim to reduce the burden of CVD. For example, long-term policy-level interventions in support of an adequate number (and quality of) educational institutions, and opportunities for education across the life course, could constitute a form of primary prevention (initiatives and actions to prevent risk factors associated with the development of disease). It is likely that such social engineering would have additional, spin-off benefits similarly supportive of reduced risk. In contrast, our observation that individual-level socioeconomic status is associated with lower pulse pressure lends itself to secondary prevention (early diagnosis and treatment of risk and disease). In this latter case, knowledge by medical personnel of how socioeconomic status predicts higher pulse pressure in treated hypertensive individuals suggests a need for greater attention to patient compliance with treatment regimes, tailored to level of socioeconomic status.

## 5. Strengths and Limitations

This study, which draws on a large sample of Australian adults, appears to be the first report to provide a comprehensive analysis of the social determinants of pulse pressure by examining the associations between pulse pressure and three different measures of socioeconomic status at both individual and area levels. Given that socioeconomic status is a multidimensional construct and that different measures of socioeconomic status are likely to affect health through different causal pathways and at different levels (e.g., individual *vs.* area level), examination of three different measures of socioeconomic status at both individual and area levels are strengths of this study. This study also examined the associations between pulse pressure and measures of socioeconomic status according to hypertensive state. In so doing, we were able to determine whether a social gradient, specific to the hypertensive state, exists for pulse pressure. A benefit of this approach is that our findings are unlikely to be confounded by the association that exists between social status and hypertension.

This study has several limitations. Firstly, this study was cross-sectional in design and thus temporal direction of the relationships investigated is unclear. Secondly, individual-level measures of education and employment status were expressed as dichotomised variables, possibly obscuring social gradients that may exist within these measures. Also, since we only examined the association between pulse pressure and employment status, we were unable to identify differences that may exist amongst different occupations. Given that lifestyle behaviors were self-reported, it is possible that some responses were influenced by social desirability and recall bias. Furthermore, since pulse pressure and systolic blood pressure are correlated [28,32,33], it is important to decipher whether associations between socioeconomic status and pulse pressure are independent of systolic blood pressure. To do this, analyses (not shown here) were repeated for systolic blood pressure in place of pulse pressure. These analyses revealed a different pattern of results, in that associations between pulse pressure and the different measures of socioeconomic status were stronger, more consistent and more robust with the addition of covariates. These results suggest that the associations identified in this study for pulse pressure are unlikely to be the product of a correlation between pulse pressure and systolic blood pressure. Additionally, although we controlled for the use of medication and stratified participants according to hypertensive state, we did not have adequate statistical power to further stratify normotensive participants from participants with controlled hypertension. Thus, it is plausible that different associations may exist between different measures of socioeconomic status and pulse pressure for normotensive participants and controlled hypertensive participants. It must also be acknowledged that since the operationalization of spatial units for studying area effects on health remains a conceptual and methodological challenge [75], it is possible that associations identified in this study may be sensitive to the spatial unit used for analysis. Finally, our analyses accounted for clustering of observations by suburb, a predetermined administrative spatial unit. This approach, although widespread in health and place research, fails to fully account for spatial autocorrelation of observations.

## 6. Conclusion

This study provides important insight into the social determinants of pulse pressure, an important cardiovascular risk factor. Our analyses reveals area-level education and individual-level income and employment status are significant predictors of pulse pressure in normotensive individuals, whereas only individual-level measures of socioeconomic status were significant predictors of pulse pressure in the hypertensive group. The findings of our study suggest area-level education may reflect underlying, unmeasured, risk factors or determinants of health that might further protect normotensive individuals from cardiovascular risk.

## References

[1] Clark A., DesMeules M., Luo W., Duncan A., Wielgosz A. (2009). Socioeconomic status and cardiovascular disease: Risks and implications for care. Nat. Rev. Cardiol..

[2] Kaplan G., Keil J. (1993). Socioeconomic factors and cardiovascular disease: A review of the literature. Circulation.

[3] Albert M., Glynn R., Buring J., Ridker P. (2006). Impact of traditional and novel risk factors on the relationship between socioeconomic status and incident cardiovascular events. Circulation.

[4] Conen D., Glynn R., Ridker P., Buring J., Albert M. (2009). Socioeconomic status, blood pressure progression, and incident hypertension in a prospective cohort of female health professionals. Eur. Heart J..

[5] Kanjilal S., Gregg E., Cheng Y., Zhang P., Nelson D., Mensah G., Beckles G. (2006). Socioeconomic status and trends in disparities in 4 major risk factors for cardiovascular disease among US adults, 1971–2002. Arch. Intern. Med..

[6] Luepker R., Rosamond W., Murphy R., Sprafka J., Folsom A., McGovern P., Blackburn H. (1993). Socioeconomic status and coronary heart disease risk factor trends. The Minnesota Heart Survey. Circulation.

[7] Dyer A., Stamler J., Shekelle R., Schoenberger J. (1976). The relationship of education to blood pressure: findings on 40,000 employed Chicagoans. Circulation.

[8] Garrison R., Gold R., Wilson P., Kannel W. (1993). Educational attainment and coronary heart disease risk: The Framingham Offspring Study. Prev. Med..

[9] Diez Roux A., Merking S., Arnett D., Chambless L., Massing M., Nieto J., Sorlie P., Szklo M., Tyroler H., Watson R. (2001). Neighborhood of residence and incidence of coronary heart disease. N. Engl. J. Med..

[10] Diez-Roux A., Nieto J., Muntaner C., Tyroler H., Comstock G., Shahar E., Cooper L., Watson R., Szklo M. (1997). Neighborhood environments and coronary heart disease: A multilevel analysis. Am. J. Epidemiol..

[11] Abeyta I.M., Tuitt N.R., Byers T.E., Sauaia A. (2012). Effect of community affluence on the association between individual socioeconomic status and cardiovascular disease risk factors, Colorado, 2007–2008. Prev. Chronic. Dis..

[12] Chaix B., Bean K., Leal C., Thomas F., Havard S., Evans D., Jégo B., Pannier B. (2010). Individual/neighborhood social factors and blood pressure in the RECORD Cohort Study: Which risk factors explain the associations?. Hypertens.

[13] Stander J., Stander R., Neaton J.D. (1993). Blood pressure, systolic and diastolic, and cardiovascular risks: US population data. Arch. Intern. Med..

[14] Kannel W.B. (1996). Blood pressure as a cardiovascular risk factor: Prevention and treatment. JAMA.

[15] MacMahon S., Peto R., Cutler J., Collins R., Sorlie P., Neaton J., Abbott R., Godwin J., Dyer A., Stamler J. (1990). Blood pressure, stroke, and coronary heart disease. Part 1. Prolonged differences in blood pressure: Prospective observational studies corrected for the regression dilution bias. Lancet.

[16] Neaton J., Wentworth D. (1992). Serum cholesterol, blood pressure, cigarette smoking, and death from coronary artery disease: Overall findings and differences by age for 316,099 white men. Arch. Intern. Med..

[17] van den Hoogen P., Feskens E., Nagelkerke N., Menotti A., Nissinen A., Kromhout D. (2000). The relation between blood pressure and mortality due to coronary heart disease among men in different parts of the world. N. Engl. J. Med..

[18] Nogueira A., Marcopito L., Lanas F., Galdames D., Jialiang W., Jing F., Hu F., Ruiz A., Lamsudin R., Beltran A., Chongsuvivatwong V., Poungvarin N., Kanluan T., Tatsanavivat P., Heller R., O’Connell D., Dobson A., Tiessens L. (1992). Risk factors for cardiovascular disease in the developing world. A multicentre collaborative study in the International Clinical Epidemiology Network (INCLEN). J. Clin. Epidemiol..

[19] Avendano M., Kunst A., Huisman M., Lenthe F.V., Bopp M., Regidor E., Glickman M., Costa G., Spadea T., Deboosere P., Borrell C., Valkonen T., Gisser R., Borgan J.-K., Gadeyne S., Mackenbach J.P. (2006). Socioeconomic status and ischaemic heart disease mortality in 10 western European populations during the 1990s. Heart.

[20] Matheson F., White H., Moineddin R., Dunn J., Glazier R. (2010). Neighbourhood chronic stress and gender inequalities in hypertension among Canadian adults: A multilevel analysis. J. Epidemiol. Community Health.

[21] Chaix B., Ducimetière P., Lang T., Haas B., Montaye M., Ruidavets J., Arveiler D., Amouyel P., Ferrières J., Bingham A., Chauvin P. (2008). Residential environment and blood pressure in the PRIME Study: Is the association mediated by body mass index and waist circumference?. J. Hypertens.

[22] Cozier Y., Palmer J., Horton N., Fredman L., Wise L.A., Rosenberg L. (2007). Relation between neighborhood median housing value and hypertension risk among black women in the United States. Am. J. Public Health.

[23] Lawes C., Vander Hoorn S., Rodgers A. (2008). Global burden of blood-pressure-related disease, 2001. Lancet.

[24] Dorjgochoo T., Shu X., Zhang X., Li H., Yang G., Gao L., Cai H., Gao Y., Zheng W. (2009). Relation of blood pressure components and categories and all cause, stroke and coronary heart disease mortality in urban Chinese women: A population-based prospective study. J. Hypertens.

[25] Mattace-Raso F., van der Cammen T., van Popele N., van der Kuip D., Schalekamp M., Hofman A., Breteler M., Witteman J. (2004). Blood pressure components and cardiovascular events. J. Am. Geriatr. Soc..

[26] Assmann G., Cullen P., Evers T., Petzinna D., Schulte H. (2005). Importance of arterial pulse pressure as a predictor of coronary heart disease risk in PROCAM. Eur. Heart J..

[27] Franklin S., Khan S., Wong N., Larson M., Levy D. (1999). Is pulse pressure useful in predicting risk for coronary heart disease? The Framingham heart study. Circulation.

[28] Panagiotakos D., Kromhout D., Menotti A., Chrysohoou C., Dontas A., Pitsavos C., Adachi H., Blackburn H., Nedeljkovic S., Nissinen A. (2005). The relation between pulse pressure and cardiovascular mortality in 12,763 middle-aged men from various parts of the world: A 25-year follow-up of the seven countries study. Arch. Intern. Med..

[29] Regidor E., Banegas J., Gutiérrez-Fisac J., Domínguez V., Rodríguez-Artalejo F. (2006). Influence of childhood socioeconomic circumstances, height, and obesity on pulse pressure and systolic and diastolic blood pressure in older people. J. Hum. Hypertens.

[30] Banegas J., Rodríguez-Artalejo F., Ruilope L., Graciani A., Luque M., de la Cruz-Troca J., García-Robles R., Tamargo J., Rey-Calero J. (2002). Hypertension magnitude and management in the elderly population of Spain. J. Hypertens.

[31] Rogers R.G., Saint Onge J. (2005). Race/ethnic and sex differentials in pulse pressure among US Adults. Ethn. Dis..

[32] García-Palmieriemail M., Crespoemail C., Mc Gee D., Sempos C., Smit E., Sorlie P. (2005). Wide pulse pressure is an independent predictor of cardiovascular mortality in Puerto Rican men. Nutr. Metab. Cardiovasc. Dis..

[33] Millar A., Lever A., Burke V. (1999). Pulse pressure as a risk factor for cardiovascular events in the MRC Mild Hypertension Trial. J. Hypertens.

[34] Benetos A., Safar M., Rudnichi A., Smulyan H., Richard J., Ducimetiere P., Guize L. (1997). Pulse pressure: A predictor of long-term cardiovascular mortality in a French male population. Hypertens.

[35] Benetos A., Rudnichi A., Safar M., Guize L. (1998). Pulse pressure and cardiovascular mortality in normotensive and hypertensive subjects. Hypertens.

[36] Alderman M., Cohen H., Madhavan S. (1998). Distribution and determinants of cardiovascular events during 20 years of successful antihypertensive treatment. J. Hypertens.

[37] Grant J., Taylor A., Ruffin R., Wilson D., Phillips P., Adams R., Price K. (2009). Cohort profile: The North West Adelaide Health Study (NWAHS). Int. J. Epidemiol..

[38] Australian Bureau of Statistics (2001). South Australia (SSC) Usual Resident Profile: Table U01 Usual Resident Characteristics, Cat. No. 2004.0.

[39] Grant J., Chittleborough C., Taylor A., Dal Grande E., Wilson D., Phillips P., Adams R., Cheek J., Price K., Gill T., Ruffin R. (2006). North West Adelaide Health Study Team The North West Adelaide Health Study: Detailed methods and baseline segmentation of a cohort for selected chronic diseases. Epidemiol Perspect Innov.

[40] International Diabetes Federation (IDF) International Diabetes Federation: The IDF Consensus Worldwide Definition of the Metabolic Syndrome. www.idf.org/webdata/docs/Metabolic_syndrome_definition.pdf.

[41] Wing L., Reid C., Ryan P., Beilin L., Brown M., Jennings G., Johnston C., McNeil J., Marley J., Morgan T., Shaw J., Steven I., West M. (1997). Second Australian national blood pressure study (ANBP2): Australian comparative outcome trial of ACE inhibitor- and diuretic-based treatment of hypertension in the elderly. Clin. Exp. Hypertens.

[42] Australian Bureau of Statistics (2006). Statistical Geography Volume 2 Census Geographic Areas Australia 2006, Cat. No. 2905.0.

[43] Montgomery S.M., Berney L.R., Blane D. (2000). Prepubertal stature and blood pressure in early old age. Arch. Dis. Child..

[44] Langenberg C., Hardy R., Kuh D., Wadsworth M.E. (2003). Influence of height, leg and trunk length on pulse pressure, systolic and diastolic blood pressure. J. Hypertens.

[45] Dart A., Kingwell B.A. (2001). Pulse pressure: A review of the mechanisms and clinical relevance. J. Am. Coll. Cardiol..

[46] Gunnell D., Smith G., Frankel S., Kemp M., Peters T. (1998). Socio-economic and dietary influences on leg length and trunk length in childhood: A reanalysis of the Carnegie (Boyd Orr) survey of diet and health in prewar Britain (1937–1939). Paediatr. Perinat. Epidemiol..

[47] Tanaka H., Dinenno F., Monahan K., Clevenger C., DeSouza C., Seals D. (2000). Aging, habitual exercise, and dynamic arterial compliance. Circulation.

[48] Stringhini S., Sabia S., Shipley M., Brunner E., Nabi H., Kivimaki M., Singh-Manoux A. (2010). Association of socioeconomic position with health behaviors and mortality. JAMA.

[49] Australian Bureau of Statistics (2009). National Health Survey: Users’ Guide—Electronic Publication, 2007–2008, Cat. No. 4363.0.55.001.

[50] Ainsworth B.E., Haskell W.L., Whitt M.C., Irwin M.L., Swartz A.M., Strath S.J., Leon A.S. (2000). Compendium of physical activities: An update of activity codes and MET intensities. Med. Sci. Sports Exerc..

[51] National Health and Medical Research Council Australian Guidelines to Reduce Health Risks from Drinking Alcohol. 2012. www.nhmrc.gov.au/_files_nhmrc/file/publications/synopses/ds10-alcohol.pdf.

[52] Hubbard A.E., Ahern J., Fleischer N.L., Laan M.V., Lippman S.A., Jewell N., Satariano W.A. (2010). To GEE or not to GEE: Comparing population average and mixed models for estimating the associations between neighborhood risk factors and health. Epidemiology.

[53] Ashe M., Miller W., Eng J., Noreau L. (2009). Physical activity and chronic conditions research team older adults, chronic disease and leisure-time physical activity. Gerontology.

[54] Siahpush M., Borland R. (2001). Socio-demographic variations in smoking status among Australians aged ≥18: Multivariate results from the 1995 National Health Survey. Aust. N. Z. J. Public Health.

[55] Panditemail A., Tang J., Bailey S., Davis T., Bocchini M., Persell S., Federman A., Wolf M. (2009). Education, literacy, and health: Mediating effects on hypertension knowledge and control. Prev. Chronic. Dis..

[56] van Rossum C., van de Mheen H., Witteman J., Hofman A., Mackenbach J., Grobbee D. (2000). Prevalence, treatment, and control of hypertension by sociodemographic factors among the Dutch elderly. Hypertens.

[57] Crespo C., Ainsworth B., Keteyian S., Heath G., Smit E. (1999). Prevalence of physical inactivity and its relation to social class in U.S. adults: Results from the Third National Health and Nutrition Examination Survey, 1988–1994. Med. Sci. Sports Exerc..

[58] Huisman M., Kunst A., Mackenbach J. (2005). Inequalities in the prevalence of smoking in the European Union: Comparing education and income. Prev. Med..

[59] Colhoun H., Hemingway H., Poulter N. (1998). Socio-economic status and blood pressure: An overview analysis. J. Hum. Hypertens.

[60] Naimi A., Paquet C., Gauvin L., Daniel M. (2009). Associations between area-level unemployment, body mass index, and risk factors for cardiovascular disease in an urban area. Int. J. Environ. Res. Public Health.

[61] Alder N., Newman K. (2002). Socioeconomic disparities in health: Pathways and policies. Health Aff..

[62] Braveman P.A., Cubbin C., Egerter S., Chideya S., Marchi K., Metzler M., Posner S.  (2005). Socioeconomic status in health research: One size does not fit all. JAMA.

[63] Ross C., Wu C. (1995). The links between education and health. Am. Sociol. Rev..

[64] Adler N., Newman K. (2002). Socioeconomic disparities in health: Pathways and policies. Health Aff..

[65] Ross C., Mirowski J. (1995). Does employment affect health?. J. Health Soc. Behav..

[66] Bartley M. (1994). Unemployment and ill health: Understanding the relationship. J. Epidemiol. Community Health.

[67] Shah D. Healthy worker effect phenomenon. Indian J. Occup. Environ. Med..

[68] Daniel M., Moore S., Kestens Y. (2008). Framing the biosocial pathways underlying associations between place and cardiometabolic disease. Health Place.

[69] Daniel M., Kestens Y., Paquet C. (2009). Demographic and urban form correlates of healthful and unhealthful food availability in Montréal, Canada. Can. J. Public Health.

[70] Kestens Y., Daniel M. (2010). Social inequalities in food exposure around schools in an urban area. Am. J. Prev.e Med..

[71] Reidpath D.D., Burns C., Garrard J., Mahoney M., Townsend M. (2002). An ecological study of the relationship between social and environmental determinants of obesity. Health Place.

[72] Ross C.E. (2000). Walking, exercising, and smoking: Does neighborhood matter?. So. Sci. Med..

[73] Yen I.H., Kaplan G.A. (1998). Poverty area residence and changes in physical activity level: Evidence from the Alameda County Study. Am. J. Public Health.

[74] Sundquist K., Theobald H., Yang M., Li X., Johansson S., Sundquist J. (2006). Neighborhood violent crime and unemployment increase the risk of coronary heart disease: A multilevel study in an urban setting. Soc. Sci. Med..

[75] Riva M., Apparicio P., Gauvin L., Brodeur J. (2008). Establishing the soundness of administrative spatial units for operationalising the active living potential of residential environments: An exemplar for designing optimal zones. Int. J. Health Geogr..

